# Quality control and evaluation of plant epigenomics data

**DOI:** 10.1093/plcell/koab255

**Published:** 2021-10-14

**Authors:** Robert J Schmitz, Alexandre P Marand, Xuan Zhang, Rebecca A Mosher, Franziska Turck, Xuemei Chen, Michael J Axtell, Xuehua Zhong, Siobhan M Brady, Molly Megraw, Blake C Meyers

**Affiliations:** 1 Department of Genetics, University of Georgia, Athens, Georgia 30602, USA; 2 School of Plant Sciences, University of Arizona, Tucson, Arizona 85721, USA; 3 Department of Plant Developmental Biology, Max Planck Institute for Plant Breeding Research, Köln, Germany; 4 Department of Botany and Plant Sciences, University of California, Riverside, California 92521, USA; 5 Department of Biology and Huck Institutes of the Life Sciences, The Pennsylvania State University, University Park, Pennsylvania 16801, USA; 6 Wisconsin Institute for Discovery & Laboratory of Genetics, University of Wisconsin-Madison, Madison, Wisconsin 53715, USA; 7 Department of Plant Biology and Genome Center, University of California Davis, Davis, California 95616, USA; 8 Department of Botany and Plant Pathology, Center for Quantitative Life Sciences, Oregon State University, Corvallis, Oregon 97331 USA; 9 Donald Danforth Plant Science Center, St Louis, Missouri 63132, USA; 10 Division of Plant Sciences, University of Missouri, Columbia, Missouri 65211, USA

## Abstract

Epigenomics is the study of molecular signatures associated with discrete regions within genomes, many of which are important for a wide range of nuclear processes. The ability to profile the epigenomic landscape associated with genes, repetitive regions, transposons, transcription, differential expression, cis-regulatory elements, and 3D chromatin interactions has vastly improved our understanding of plant genomes. However, many epigenomic and single-cell genomic assays are challenging to perform in plants, leading to a wide range of data quality issues; thus, the data require rigorous evaluation prior to downstream analyses and interpretation. In this commentary, we provide considerations for the evaluation of plant epigenomics and single-cell genomics data quality with the aim of improving the quality and utility of studies using those data across diverse plant species.

## Teaser

Useful consideration for quality control and evaluation of plant epigenomics data are provided.

## Introduction

High-throughput sequencing has revolutionized the study of plant epigenomes. The cost of sequencing has never been cheaper, which has shifted the bottleneck to sample collection, epigenomic assays, sequencing library preparation, and data analysis. Plant samples are readily evaluated for transcript and small RNA (smRNA) abundance, as well as the patterns and distribution of transcription factor (TF) binding sites, DNA methylation, histone modifications, and chromatin accessibility. Some of these assays have even been adopted for use in single cells. However, with the democratization of sequencing, challenges arise with regard to the proper evaluation of experimental and data quality. Data quality can have numerous definitions, but here we use this term to refer to the quality and outcome of the overall experiment and its usefulness for uncovering biological phenomena and not to the “sequencing quality” scores generated by sequencing instruments. The aim of this commentary is to raise awareness of data quality standards, define common pitfalls and issues associated with large-scale experiments, and provide considerations that we suggest colleagues embrace when generating and analyzing plant epigenomics data.

What distinguishes transcriptome and epigenome assays from genome sequencing is biological (as opposed to genotypic) and experimental variability (dependent on endogenous and exogenous stimuli as well as the complexity of the assay itself). As a result, whole-genome sequencing (WGS) typically does not require biological replicates, whereas the quantitative nature of transcriptomics and epigenomics typically does. For example, for genome sequencing, the identification of single-nucleotide (nt) polymorphisms can be viewed as a qualitative difference at a single position between two genotypes. Given sufficient sequencing depth and uniform genome coverage, these binary differences between genotypes are readily identified in the absence of replication. However, comparisons of transcript or smRNA abundance between samples are often used in a quantitative manner and, as a result, must be accompanied by biological replication to support the major claims.

Replicates from distinct biological samples are required to exclude technical and biological variance not associated with the variable of interest, especially in the context of studies that draw conclusions based on quantitative changes in gene expression (Schurch et al., 2016). Technical replicates (within an experiment) provide insight into variation associated with an assay, but they do not describe the range of results expected within a population (Vaux et al., 2012). While unreplicated studies have been published for many types of “-seq” data, such as single-cell transcriptome sequencing and early studies of chromatin immunoprecipitation (ChIP) coupled with sequencing, most fields have matured to the point in which the need for proper biological replication outweighs the “wow factor” of the new technology; similar shifts have occurred going back to microarray studies, RNA-seq, and a wide variety of “-seq” studies. That is, the first few papers describing a new technology were often accepted with minimal biological replication, because the method is novel, likely to be of high value to the scientific community, and therefore deemed worthy of rapid publication. Scientific advances often require a delicate balance between the development and application of new methods. In the early days of most technologies and data-rich approaches, costs are high. However, at a certain point, as the approach becomes more routine for biological exploration and the associated costs (typically) decline, the essential components of good experimental design, including biological replication, must come to the fore.

## Evaluation of enrichment and additional quality controls

A distinguishing feature of transcriptome/epigenome sequencing approaches compared to genome sequencing is the reliance on the enrichment of molecules from a larger pool, often using biochemical approaches. For example, most RNA-sequencing (RNA-seq) experiments rely on the enrichment of mRNA from total RNA by the capture of mRNA via poly(A) tails. For smRNAs, size exclusion is used as a form of enrichment. To detect TF binding or histone modification abundance using ChIP-sequencing (ChIP-seq), antibodies are required to capture specific protein : DNA interactions. Similarly, the detection of accessible chromatin requires the isolation of chromatin, followed by enzymatic treatment to enrich nucleosome-depleted regions. The need to enrich for RNA, smRNA, and/or chromatin increases the complexity of these assays, resulting in an increase in both technical and biological variability in the data linked to the assay materials (i.e. kit components, chemicals, or plant tissue quality), the individual experimenter(s), or the location where the experiment was performed.

In this commentary, we provide some useful considerations for replication and quality control metrics that can be used to evaluate enrichment/data quality for common transcriptome and epigenome assays (Table  1). The evaluation of these metrics will help researchers determine if experiments should be repeated or if caution should be taken with interpretation of the data. Adding quality control metrics to supplemental material is commonly accepted by journals for some assays, yet lacking for others. We strongly believe that including some of these quality control metrics for high-throughput sequencing-based experiments will improve the ability of readers to accurately evaluate the results both pre and postpublication. We describe several issues that are common among multiple types of assay, followed by a description of data type-specific considerations.

**Table 1:** Recommendations for important checks and controls for plant epigenome assays

Important Checks and Controls	RNA-seq	smRNA-seq	WGBS	ChIP-seq	Chromatin Accessibility	Single-cell RNA-seq	Single-cell ATAC-seq
Low level of duplicate reads	x	x	x	x	x	x	x
Appropriate normalization	x	x	x	x	x	x	x
Report number of sequenced and aligned reads	x	x	x	x	x	x	x
Show representative genome browser screen shots (including replicates)	x	x	x	x	x	x	x
Evaluate RNA/DNA quality	x	x	x				
High and/or consistent alignment rates	x	x	x	x	x	x	x
Show Venn diagram or Upset plots of identified clusters/regions/peaks between replicates		x	x	x	x		
Aligned smRNA sequences are enriched for 21–24 nt sizes		x					
Report bisulfite conversion rates			x				
Report read coverage of the genome			x				
Report SPOT or FRiP scores				x	x		x
Implement IDR with replicates				x			
Include input or IP background control				x			
Plot read coverage around genomic features (genes/TEs/TSSs, etc.)				x	x		x
Consider a spike-in control		x		x			
Evaluate enzymatic bias using genomic DNA control					x		
Report number of cells targeted						x	x
Report number of unique transcripts/Tn5 integrations per cell						x	x
Evaluate marker genes						x	x
Filter cells with high proportion of organellar reads							x

### PCR bias

When possible, the amount of input material used for each epigenomic library should be standardized to reduce variation that can be attributed to polymerase chain reaction (PCR) bias. The number of PCR cycles should be minimized to reduce PCR bias and duplicate reads. Duplicate reads are commonly used as a measure of library quality and complexity. A duplicate read is one that has the same start and end point and is often the result of PCR duplication as opposed to an independent biological event. In-depth analyses of sequenced libraries require careful inspection of the level of enrichment achieved. Unfortunately, although there are methods to evaluate enrichment and data quality, they are rarely included in the presentation of data in manuscripts, which shifts the burden of evaluating data quality to the reader or user, postpublication.

### Sequence alignments

For all transcriptome and epigenome data types generated from sequencing, it is important to have an understanding of the limitations of sequence alignments. There are numerous strategies for the alignment of sequenced reads, depending on the scientific question being pursued as well as downstream analyses and data interpretation. Arguably, just as important is an understanding of the limitations of current genome assemblies and annotations as well as computational packages designed to work with well-assembled genomes. We are fortunate to be able to conduct these experiments in an era when high-quality reference genomes exist for many plants, and new assemblies and versions are continually being released. However, even for these high-quality genomes, assembly and annotation errors still exist that can lead to erroneous results (Mendieta et al., 2021).

### Challenges with “peak” identification

Analysis of transcriptomic and epigenomic data requires dedicated pipelines for which the subject of the analysis dictates how the data should be processed. For example, in RNA-seq, comparisons are typically made at the level of transcripts or gene loci, which provides a fixed set of regions that are generally uniquely mapped and supported by prior analysis and data, such as genome assembly, annotation, cDNA support from Expressed Sequence Tags, and previous RNA-seq data. Analysis of epigenomic data is typically more challenging, given that regions of sequencing coverage enrichment (i.e. regions that resolve into peaks) have rarely been preidentified using a gold standard approach and can be associated with repetitive DNA regions that are difficult to assemble. That is, an epigenomic experiment may identify novel peaks that were not previously observed and reported, thus making it challenging to cross-check or validate those results using existing data.

**Figure 1:**
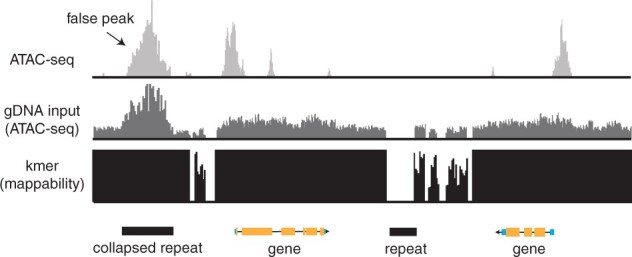
Accounting for mappability and genome assembly artifacts. A schematic diagram of typical aligned data obtained by ATAC-seq. Regions of chromatin accessibility are indicated by peaks. A region that appears enriched for sequencing coverage (labeled “false peak”) is actually due to a collapsed repeat in the genome assembly. Tn5-treated genomic DNA helps to identify these problematic regions. A k-mer-based approach is used to reveal regions of the genome that are uniquely mappable for a given sequence fragment length.

Another major consideration is the evaluation of peak quality and quantity between samples. The main goal of smRNA-seq, ChIP-seq, and chromatin accessibility mapping experiments is to identify regions in the genome that are enriched for biological signals (i.e. have a significant density of stacked sequenced reads; Figure  1). Upon sequencing, these regions are often referred to as peaks; an array of software packages have been designed to identify these regions in a genome wide-fashion (Ji et al., 2008; Zhang et al., 2008; Rozowsky et al., 2009; Heinz et al., 2010; Guo et al., 2012; Xu et al., 2014; Ibrahim et al., 2015; Meers et al., 2019). These software packages perform signal processing steps in an attempt to approximate human judgment and generally involve smoothing and cutoff parameters. Given the wide variety of signal shapes that can occur within and between data types, there is no gold standard for these parameter selections. However, when biological replicates are available, there are recommended procedures to perform after a peak caller is applied. When biological replicates are sequenced, correlations between datasets can be used to evaluate data quality and to estimate high-confidence target peaks. For example, it is common practice to use correlation analyses between biological replicates to evaluate RNA-seq samples. Comparisons of genome-wide transcript abundance from entire organs for each gene often reveal correlations ˃0.95. However, for epigenomic assays, such as ChIP-seq and chromatin accessibility mapping, correlations of reads in peaks (as opposed to genes) should be used to evaluate consistency between biological replicates, because read coverage of large predefined genomic bins (e.g. 10 or 100 kilobase [kb]) obfuscates technical and experimental variation.

Another method used to evaluate peak quality and sample-to-sample variation involves the irreproducible discovery rate (IDR; Li et al., 2011). This method uses a statistical framework to compare the signal ranks of peaks, providing IDR values for all peaks across biological replicates. A comparison of the number of peaks passing the determined IDR threshold between true and re-sampled pseudo-replicates allows the numeric quality of replicated enrichment experiments to be assessed. According to the standards set by the ENCODE project, the ratio between peaks enriched in replicates and pseudo-replicates should be between 0.5 and 2.0 (Landt et al., 2012). A large proportion of peaks above the IDR threshold indicate substantial discrepancies between signals among replicates, which is indicative of poor reproducibility. We note that large variation in the number of sequencing reads between biological replicates can result in variable “peak” detection due to the sensitivity of identification of rare or cell-type-specific peaks (Jung et al., 2014).

### Controls that account for assembly quality and the sequence alignment strategy

Some of the most challenging-to-study regions of any genome are the repeats (e.g. transposons [TEs] or centromeric regions), as they confound most assembly algorithms and are often collapsed into single units in assemblies, even though they exist as complex arrays in the genome. Due to their repetitive nature, these regions often appear as “peaks” in any coverage-based assay such as ChIP-seq and chromatin accessibility mapping, even if the actual result is not an enrichment or peak. In other words, the collapse of the repeats in the genome assembly can yield misleading artifacts. Peaks occurring as a result of a genome assembly or sequence alignment strategy obscure true peaks that result from biological signals. One strategy to evaluate the sequence alignment approach and to manage these problematic regions of the genome is to determine sequence mappability using a k-mer-based approach that matches the read length from a library (Derrien et al., 2012). An alternative approach is to compare these problematic regions that result from mis-assembly or repeat regions to a genomic DNA sequencing library, such as WGS or ChIP-seq/chromatin accessibility mapping input (Figure  1). Next, for coverage-based enrichment assays, determine how many “peaks” are identified from the genomic DNA library (Figure  1). Any peaks found in this control library within mappable regions of the genome are likely artifacts of the alignment strategy and/or the genome assembly (Figure  1; Pickrell et al., 2011). An alternative approach is to use the input or WGS alignments as a baseline to measure enrichment (Figure  1). By knowing which regions of the genome behave in this manner, those regions can be excluded from downstream analyses, reducing associated erroneous results.

### Data visualization

A valuable tool that should be used to quickly assess the quality of transcriptomic and epigenomics data is a genome browser, such as the Integrative Genomics Viewer (Thorvaldsdottir et al., 2013) or JBrowse (Buels et al., 2016; Hofmeister and Schmitz, 2018). These genome browsers allow aligned data and/or processed files to be visualized. The visualized data can be used to roughly evaluate the uniformity of coverage or the enrichment of transcripts, smRNAs, DNA methylation, or peaks around expected genomic regions. The data can also be used to evaluate the accuracy of software used to identify differentially methylated regions and/or differential peaks. It should be noted that genome browsers and associated screenshots do not supplant quantitative measurements of the data, but they are important tools for data presentation and are recommended for the additional evaluation of data quality.

## Data type-specific considerations

### RNA-seq

There are numerous applications of RNA-seq technologies, but in this section, we will focus specifically on the sequencing of mRNAs. Quantitative and qualitative measurement of RNA is a cornerstone of genome biology. Measuring mRNAs is the most common: the data are used to annotate genomes, discover splice isoforms, and most often to estimate steady-state transcript levels. The estimates of transcript levels are typically compared across treatments, genotypes, or tissue types to infer or determine which genes contribute to key differences in the compared materials.

#### Important checks and controls

First and foremost, RNA integrity should be evaluated prior to library preparation (e.g. with a fragment bioanalyzer), as poor-quality RNA can lead to erroneous artifacts. In most cases, biological triplicates are sufficient for the identification of differentially expressed genes, although the number can vary depending on the study system, the question being posed, and the sensitivity needed from the analysis. Key indicators of high-quality RNA-seq libraries include high and consistent sequence alignment percentages, low levels of duplicate reads, and uniform distribution of reads across transcript annotations. Low or inconsistent alignment percentages between samples likely indicate a problem with RNA quality. These types of libraries often possess many more nonpoly(A)-tailed RNAs compared to high-quality libraries. One emerging method that is useful for evaluating duplicate reads is to use unique molecular identifiers, which are commonly used in single-cell genomics. In parallel, incorporating spike-in RNAs is useful for estimating transcript abundance. A variety of methods are used to normalize RNA-seq data, but the two main approaches are normalizing by library size and transcript length or implementing a quantile normalization approach. The latter is typically used when changes in transcript abundance are not normally distributed.

### smRNA-seq

Plant smRNAs (here defined as RNAs produced by Dicer-like [DCL] proteins and bound to Argonaute [AGO] effector proteins) are categorized into discrete classes based on their size (generally 21- 24-nt long) and mode of action. The two major classes are small interfering RNAs (siRNAs) and microRNAs (miRNAs). The functions of siRNAs in RNA-directed DNA methylation (to silence repeats and TEs) and miRNAs or secondary siRNAs in the posttranscriptional regulation of mRNAs have been extensively reviewed (Axtell, 2013a; Holoch and Moazed, 2015).

#### Important checks and controls

Upon sequence alignment, the majority of DCL/AGO-related smRNAs from most plants range between 21- and 24-nt long. The absence of discernible peaks of 21- and/or 24-nt RNAs in the total size distribution of a smRNA-seq alignment could indicate issues with the quality of the input RNA or library (Mathioni et al., 2017). smRNA library preparation is often accompanied by a size exclusion step to separate these RNAs from mRNAs, tRNAs, and rRNAs. However, poor RNA quality can lead to the degradation of these longer RNAs, which leads to their abundance within fractionations of smRNA populations. These RNAs can easily be filtered, especially for tRNAs and rRNAs; however, degraded mRNAs are more challenging to filter, as genes are often potential targets of the smRNAs under investigation. Therefore, to evaluate if an mRNA is a true source of siRNAs, the strandedness and size of the aligned RNAs can be examined. For example, a sign of mRNA degradation versus true smRNAs is the presence of numerous sequenced reads that are outside the expected size ranges (21–24 nt) that are derived primarily from the sense strand of the transcript. Computational analyses can be performed to identify individual loci where aligned smRNAs are not predominantly in the 21–24 nt size range (Axtell, 2013b; Johnson et al., 2016). A handful of such loci does not necessarily indicate a fatal flaw in the library; some mRNAs, especially highly abundant ones, likely have a true in vivo population of semi-degraded fragments.

Other possible issues include amplification bias, which is observed as a paucity of distinct or unique reads; this is most commonly observed when the input quantities of RNA are well below the levels recommended for the protocol (Wright et al., 2019). Differential expression of smRNAs using sequencing should require a minimum of three biological replicates and the use of proper statistical procedures that robustly model dispersion and control the false discovery rate (Schurch et al., 2016). Additionally, the choice of an appropriate normalization method is critical when quantifying smRNAs and identifying differentially accumulated smRNAs between samples. While normalization against total smRNA reads or rRNA/tRNA-filtered total smRNA reads is most common, this can generate biased results when changes in a class of smRNAs are found in a particular genotype or sample. In fact, rRNA/tRNA fragments constitute a fair proportion of total smRNA reads and can serve as an internal control for normalization (McCormick et al., 2011), provided that all samples were prepared at the same time with the same methodology and sequenced in the same sequencing run. When sRNA-seq data are to be used quantitatively, we recommend that several normalization methods be applied and the outcomes validated by qRT-PCR or northern blotting against select miRNAs or siRNAs known to be stable across all samples being analyzed.

### Cytosine DNA methylation

DNA methylation can be examined at single nt resolution using whole-genome bisulfite sequencing (WGBS) or enzymatic methyl-seq (Cokus et al., 2008; Lister et al., 2008; Feng et al., 2020). These single-base resolution technologies are especially useful in plants, as DNA methylation occurs in three distinct contexts (CG, CHG, and CHH, where H = A, C, or T) that reflect the activities of distinct DNA methylation pathways (Law and Jacobsen, 2010). DNA methylation is highly enriched at transcriptionally silent regions of plant genomes such as TEs and repeats (Cokus et al., 2008; Lister et al., 2008; Feng et al., 2010; Zemach et al., 2010; Niederhuth et al., 2016). It is also found within gene bodies of a subset of actively transcribed genes in angiosperms as well as some ferns and gymnosperm species (Tran et al., 2005; Takuno and Gaut, 2012; Takuno et al., 2016; Bewick et al., 2017). Methylome data are useful for a variety of reasons; their intended use for describing qualitative versus quantitative aspects of the samples influences the need for biological replication. For example, methylome data that accompanies a genome assembly and is used to broadly characterize and annotate the methylation patterns around genes, transposon, repeats and other genomic features doesn’t typically require replication. However, the identification of differentially methylated regions requires biological replication. Here, the analysis is often focused on comparing the same genomic region across a variety of samples derived from a different genotype (mutant or natural isolate), a different tissue, or subjected to different environmental conditions. Similar to other quantitative methods like RNA-seq, a variety of software packages are publicly available that are specifically tailored to process, align, and identify differentially methylated positions and/or regions between samples from a treatment versus control group and/or a population (Xi and Li, 2009; Krueger and Andrews, 2011; Schultz et al., 2015).

#### Important checks and controls 

Input genomic DNA used for methylome sequencing should be of high quality, although it does not need to be as pure as samples that are prepared for genome assemblies. Poor input quality of genomic DNA can lead to uneven uniformity of coverage across the genome, making comparisons between samples and biological replicates challenging. WGBS depends on either the chemical or enzymatic conversion of unmethylated cytosine to uracil (Frommer et al., 1992; Feng et al., 2020). Upon PCR, uracil is converted to thymine. An important consideration for methylome sequencing is the conversion rate of the treatment. This can be measured by evaluating the percentage of cytosines detected as “methylated” in the chloroplast genome or in an unmethylated spike-in DNA control, such as lambda phage DNA. To detect small changes in methylation, conversion rates should be ˃99%. If using unmethylated plastid genomes as controls, one should be certain to ensure that sequences are not duplicated in the nuclear genome, as this could interfere with measuring the conversion efficiency. This conversion rate, along with sequence alignment rates (most often for uniquely aligned reads), should always be reported, typically in a supplemental table. Lastly, the expected genome coverage per sample will depend on the experiment. Typically, >15× coverage per biological replicate is sufficient for most analyses, as suggested by the Human Epigenome Roadmap Consortium (http://www.roadmapepigenomics.org/protocols). As a reminder, because methylome sequencing results in strand-specific data, 15× coverage corresponds to 7.5× per strand. Measuring the percentage of duplicate reads is also useful in evaluating library complexity. For low input or low-quality genomic DNA, a higher rate of duplicate reads is often observed. In most cases, duplicate reads should be reduced to a single read for downstream analyses, and coverage estimates should be calculated after they are removed. Otherwise, the genome coverage will be artificially inflated due to technical issues.

### ChIP-seq

ChIP-seq is a useful method for evaluating the enrichment of protein–DNA interactions (Johnson et al., 2007). In this method, plant tissue is treated with formaldehyde to crosslink and preserve protein–DNA interactions. After crosslinking, antibodies or epitope tags specific to the protein or hybrid protein of interest are used to detect and isolate the protein along with the associated DNA, which is then sequenced. This assay is commonly used to detect TF DNA binding sites or locations of posttranslationally modified or variant histones. The most important consideration for ChIP-seq is recognizing that it is an enrichment assay and that the quality of the enrichment is dependent on numerous experimental factors, such as the quality of the antibody and/or the precipitation protocol, in addition to biological factors such as the tissue specificity of the signal or its response to biological variation. A key goal of this assay is to maximize the antibody-tagged DNA signal over background. In the ideal experiment, there would be no background, meaning that no sequenced reads were detected from regions of the genome that were not associated with the protein being assayed. Unfortunately, background is essentially impossible to eliminate from this procedure, and therefore it must be accounted for using an input or other control library.

**Figure 2:**
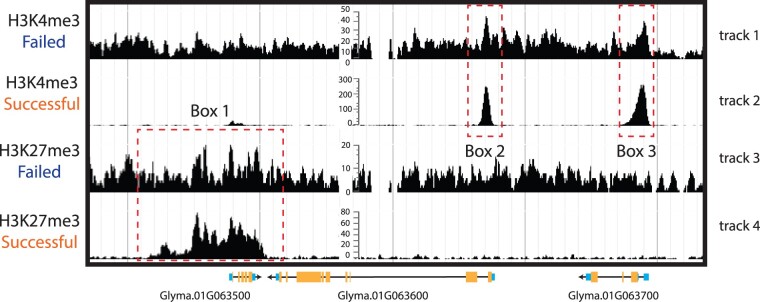
Visualization of ChIP-seq enrichment of histone modifications. Low-quality/failed versus high-quality ChIP-seq data are shown for H3K4me3 and H3K27me3 from soybean (*Glycine max*) leaves. The first and third tracks show low-quality and/or failed ChIP-seq data, whereas tracks 2 and 4 show high-quality data. Box 1 shows a region of H3K27me3 enrichment in track 4, whereas the same region shows almost no enrichment in track 3. As is typical for H3K27me3, enrichment is present throughout the gene body into the upstream region. Boxes 2 and 3 show enrichment for H3K4me3 at TSSs in track 2, whereas weak enrichment is detected in track 1.

A high-quality ChIP-seq experiment typically results in a high coverage of sequenced reads at discrete regions throughout the genome, again referred to as “peaks,” although in low-quality ChIP-seq datasets, peaks are hard to distinguish from background signal (Figure  2). The shape, location, magnitude, and sensitivity to identify peaks differ depending on the protein–DNA interaction being measured and the complexity and sequencing coverage of the sequencing library (Hower et al., 2011; Jung et al., 2014). For example, TF peaks are typically sharp and enriched near the transcriptional start site (TSS), upstream and downstream of genes, whereas histone modifications and histone variant peaks are generally broader. The patterns and distribution of histone modifications have been well characterized in certain plant genomes (Zhang et al., 2007; Bernatavichute et al., 2008; Zhang et al., 2009; Li et al., 2019; Lu et al., 2019; Ricci et al., 2019; Montgomery et al., 2020; Zhao et al., 2020). There is a high similarity in the patterns and distributions of histone modifications among plant genomes. For example, trimethylation of lysine 4 of histone 3 (H3K4me3) is highly enriched around the TSS in plant genomes, whereas H3K4me2 and H3K4me1 are found within gene bodies (Zhang et al., 2009). These expected patterns and distributions at genes or other genomic features can be used to evaluate the quality of the enrichment. A novel method referred to as Cleavage Under Targets and Release Using Nuclease or Cleavage Under Targets and Tagmentation was recently developed to measure protein–DNA interactions (Skene and Henikoff, 2017; Kaya-Okur et al., 2019). Although the method is unique, most of the same methods used for the evaluation of data quality are the same as ChIP-seq. Additionally, Tn5-based chromatin profiling methods are biased toward detecting accessible chromatin, which requires extra scrutiny in downstream analyses (Wang and Zhang, 2021).

#### Important checks and controls

Sequence alignment percentages should be evaluated and reported for each sample. The percentage of duplicate reads can be used to determine if the sample is of low complexity. This is a common issue with ChIP-seq, as sub-nanogram amounts of DNA are typically immunoprecipitated from each ChIP, especially in the case of TF mapping. The preparation of a sequencing library does not necessarily indicate that the assay worked as intended; it only indicates that input DNA was used in the library preparation. To evaluate the quality of the ChIP experiment, the enrichment of sequenced reads relative to known genomic regions such as genes, repeats, promoters, and so on should be used. Additionally, genome-wide metrics for measuring signal-to-noise, such as Signal Portion of Tags (SPOT) and Fraction of Reads in Peaks (FRiPs), are used to establish the extent of enrichment, where greater values indicate lower background (Ji et al., 2008). However, SPOT/FRiP scores are largely qualitative and dependent on the heterogeneity of the sample, varying widely depending on the species, tissue, or cell type. In cases where biological replicates are performed, IDR can be implemented to establish the reproducibility of the experiments by comparing the ratio of identified peaks between and within biological replicates (see Landt et al. (2012) for more information on IDR and recommended thresholds). It is useful to present a meta-analysis of the coverage distribution of reads across these features for all biological replicates, as well as heat maps, which reveal locus-specific patterns of enrichment. These two data types are complementary in that one provides an average score across all genomic features, whereas the other shows individual data points for the studied features. It is also recommended that authors present representative genome browser screenshots of the biologically replicated data, typically as a supplemental figure. Together, this information can be used by readers to rapidly evaluate the quality of the experiment, as strong signals are expected compared to background regions of the genome. Lastly, the goal of most ChIP-seq experiments is peak identification, which often requires an input control for comparative purposes. It is typically recommended that a portion of the isolated chromatin be used as an input control for mapping TFs. To identify peaks from histone modifications, the use of an antibody to unmodified Histone H3 is recommended, as this reflects the distribution of nucleosomes across the genome. In both cases, the input DNA can be used to control for “mappability” of the genome as described above. In certain cases, adding spike-in chromatin from another species (e.g. fruit fly [*Drosophila melanogaster*] or mouse [*Mus musculus*]) to each sample prior to performing the ChIP experiment can be useful for absolute measurements of enrichment, particularly in genomes of lesser quality. A spike-in ChIP approach is invaluable for quantifying global changes in features across biological samples, for example, when comparing a reduction in a given histone modification between strongly affected mutants and wild-type plants.

### Chromatin accessibility

Regions of the genome that are depleted of nucleosomes often reflect accessible chromatin; these regions are enriched for cis-regulatory elements (CREs) and TF binding (Stalder et al., 1980). A variety of methods are used to identify chromatin accessibility, such as micrococcal nuclease (MNase) sequencing (Liu et al., 2015; Zhang et al., 2015), DNase I sequencing (Zhang et al., 2012b; Cumbie et al., 2015), and Assay for Transposase Accessible Chromatin sequencing (ATAC-seq; Buenrostro et al., 2013; Lu et al., 2017; Maher et al., 2018). Each of these methods utilizes an enzyme that releases accessible chromatin fragments, either via enzymatic digestion (MNase or DNase) or sequencing adapter integration (ATAC-seq; Zhang et al., 2012b; Zhang et al., 2012a; Sullivan et al., 2014). These assays have been invaluable during the last decade at improving the ability to investigate the noncoding regions of the genome for candidate CREs (Rodgers-Melnick et al., 2016; Reynoso et al., 2019; Ricci et al., 2019). They are especially useful in plants with large genomes in which CREs can be located ˃100 kb away from their target gene(s) (Oka et al., 2017; Lu et al., 2019). These assays are similar to ChIP-seq in that they are evaluated based on sequencing coverage at distinct regions throughout the genome, again referred to as “peaks.” These peaks represent DNA fragments released from accessible chromatin and are highly enriched at TSSs of genes in plant genomes. However, as mentioned above, the identified regions can be proximal or distal to their target gene(s). They can even be located in introns, exons, 5′- or 3′-untranslated regions, or downstream of their target genes and interact with the target promoter through 3D chromatin interactions.

#### Important checks and controls

Unlike other epigenomic assays, accessible chromatin profiling techniques capture the signal from the ends of sequencing fragments rather than the center (e.g. ChIP-seq). As such, aligned reads from accessible chromatin profiling must be reformatted prior to the identification of peaks. For example, analysis of ATAC-seq data often includes post alignment steps that identify Tn5 integration sites at base resolution by initially collecting 5′-coordinates. These coordinates are then shifted by +5/−4 for forward and reverse alignments, respectively, to account for the 9-bp binding footprint of Tn5. As most peak callers assume that the biological signal originates from the centers of paired reads, naïve application on unprocessed chromatin accessibility sequencing data will lead to false-positive peaks, especially for sequencing libraries with generally larger insert sizes.

The percentage of aligned reads and the duplication read rate are again useful metrics. Similar to ChIP-seq, high-quality chromatin accessibility assays have a high signal-to-background ratio, often measured using FRiPs. For a detailed explanation of how to evaluate data quality from chromatin accessibility mapping assays, this article provides useful guidelines (Bubb and Deal, 2020). A key consideration is that genomic DNA should be treated with the appropriate enzyme (MNase, DNase I, or Tn5) for each new species under study to evaluate enzymatic bias and the overrepresentation of specific fragments. Another key point is that high-quality ATAC-seq data from *Arabidopsis thaliana* typically has a FRiP score >35%, whereas in maize (*Zea mays*), a sufficient score is typically >20%. The evaluation of FRiP scores is dependent on the species being studied, but in general, a higher percentage of reads in peaks reflects a higher quality experiment. FRiP scores or other metrics that evaluate signal-to-noise ratios are often influenced by the genome size and the number of sequenced reads. With a greater genome size, there is a higher probability of background reads, given that the number of chromatin-accessible regions does not scale with genome size. A sufficient number and complexity of sequenced reads should be generated such that the number of identified peaks becomes saturated. Another way to evaluate chromatin accessibility data is to plot the enrichment of read coverage around the TSSs of genes, as most expressed genes possess chromatin. If a TSS-sequencing method has not been applied to precisely identify TSS locations in a sample, they can be roughly approximated using annotated TSSs. A strong enrichment should be observed around the TSSs of genes compared to input controls. This should be performed for all biological replicates of all samples. Displaying data in a genome browser for all biological replicates are also a highly recommended approach to quickly evaluate the quality of the data and to present it to a broader audience.

### Single-cell genomics

The advent of single-cell genomics is one of the most exciting recent technological developments in genomics that will significantly influence plant biology research. The ability to survey molecular profiles of populations of individual cells from any plant species without the need to generate transgenic reporter lines will undoubtedly lead to breakthrough discoveries. Single-cell RNA-seq (scRNA-seq; Denyer et al., 2019; Jean-Baptiste et al., 2019; Ryu et al., 2019; Shulse et al., 2019; Zhang et al., 2019; Satterlee et al., 2020; Xu et al., 2021; Zhang et al., 2021b, 2021a) and single-cell ATAC-seq (scATAC-seq; Dorrity et al., 2021; Farmer et al., 2021; Marand et al., 2021) have been applied to a few plant species and tissue types. This work has thus far demonstrated the ability to resolve cell type information from bulk populations by aggregating cells of the same type, typically leveraging dimensionality reduction (principal component analysis, non-negative matrix factorization, t-distributed stochastic neighbor embedding, uniform manifold approximation and projection, etc.) and graph-based clustering techniques (Leiden and Louvain) (Blondel et al., 2008; van der Maaten and Hinton, 2008; McInnes et al., 2018; Traag et al., 2019). Single-cell methods are particularly useful for pinpointing genes expressed in a cell-type-specific manner and identifying cell-type-specific CREs. Together, scRNA-seq and scATAC-seq hold great promise for studying gene regulatory networks at cellular resolution.

There are numerous methods for preparing single-cell genomic libraries, but the most commonly used thus far in plants relies on instrumentation and reagents provided by 10× Genomics. Unlike other sequencing libraries described above, the cost of preparing single-cell libraries easily varies by 10- to-20-fold depending on the type of library. As a result, preparing the sequencing library can be more expensive than the sequencing itself, which has major implications for experimental design. Unfortunately, single-cell genomics is susceptible to both biological and technical variation, even more so than bulk cell-based assays, considering that cellular heterogeneity is masked in bulk assays by averaging signals across profiled cells. To resolve this variation, biological replicates are required, especially for studies that rely almost solely on single-cell data as the core of the story. The current standard of reproducibility for single-cell approaches relies on qualitative evaluation of replicate mixing in reduced dimensional representations (e.g. within PCA/tSNE/UMAP embeddings), or quantitatively through comparisons of cell-type proportions per biological replicate. Additional quantitative metrics of reproducibility for single-cell techniques are likely to emerge as costs continue to fall and single-cell methods become more widely adopted by the plant science community.

#### Important checks and controls

Single-cell genomic analysis results in sparse data across thousands of individual cells. As a result, single-cell genomic libraries are often sequenced to saturation, for example, more than 500 million reads per 10,000 cells/nuclei, resulting in a high rate of read duplication. The percentage of aligned sequences should be reported for all samples. Additionally, due to the single-cell resolved nature of the data, the number of target cells profiled and the number of recovered cell profiles as well as the number of processed reads per cell must be reported. For example, a typical high-quality scRNA-seq or scATAC-seq library from plants using the 10× Genomics platform will capture >40% of input cells/nuclei with at least 1,000 unique transcripts or Tn5 integrations per cell. However, lower numbers of events per cell are acceptable for species with fewer genes (e.g. the liverwort *Marchantia polymorpha*). We also recommend the quantification of ambient RNA and chromatin from lysed cells and nuclei to distinguish true cells from background noise (Young and Behjati, 2020). Additionally, scRNA-seq experiments in plants have generally relied on generating protoplasts to create single-cell suspensions. For such approaches, it is imperative to generate bulk-scale RNA-seq control datasets to account for enzyme treatment-induced changes to cell states. Caution should be taken to minimize the number of droplets and/or barcodes that possess multiple nuclei or cells, which can be evaluated empirically by genotype/species mixing (Marand et al., 2021) or through the use of cell hashing (Stoeckius et al., 2018) prior to scaling up library preparations. Additionally, for scATAC-seq, the FRiP score and enrichment of reads around the TSSs of genes can be used to evaluate the quality of the data (Marand and Schmitz, 2021). Data quality from scATAC-seq is often much better than that of bulk ATAC-seq experiments, and FRiP scores >75% and >45% have been achieved for scATAC-seq in Arabidopsis and maize, respectively (Dorrity et al., 2021; Marand et al., 2021). The exact reason for the superior data quality is not known, but the ability to exclude cells that do not have enrichment of reads around the TSS or within peaks improves downstream analyses. Lastly, cells with high amounts of reads that align to the chloroplast and/or mitochondria from scATAC-seq data can be removed, as they likely represent broken nuclei or supernatant generated from the isolation of cells. The ability to remove data from suspect cells is likely one reason for the superior data quality and improved downstream analyses using single-cell compared to bulk ATAC-seq approaches.

## Conclusions

Transcriptomics and epigenomics are revealing features of plant genomes at an unprecedented pace and providing a major source of as-yet untested hypotheses. The ability to produce high-quality sequencing libraries from these assays ranges in difficulty from relatively straightforward (RNA-seq) to challenging (ChIP-seq). However, the production of a sequencing library from any of these assays does not ensure the assay worked as intended; it only indicates that a library has been prepared and is ready for sequencing. Appropriate computational analysis of the sequenced library is required to evaluate the quality of the experiment used to produce the input RNA and/or DNA for library preparation. The major goal of this commentary is to raise awareness of the complexity of data quality issues associated with plant epigenomics research. We recognize that many of the techniques and/or considerations will need to be modified or adapted over time as novel technological and/or computational/statistical methods are developed. The considerations presented above are common methods that are currently used by many in the field to assess experiment and data quality. Presenting these key metrics as supplemental data during manuscript submission and publication will assist in the reader’s ability to appropriately evaluate interpretations pre and postpublication. Finally, just as the range of “-seq” experiments has exploded over the last decade, along with the ways to isolate tissues or cells for these methods, there are surely many new data types and acquisition approaches yet to emerge. These are likely to come with their own pitfalls, analysis quirks, and statistical challenges. We hope that authors will rise to the occasion, deeply probing and questioning the quality of their data and including their tests, concerns, and conclusions within their manuscript files to assist readers in understanding the limits of their observations and claims.

## Notes

The author responsible for distribution of materials integral to the findings presented in this article in accordance with the policy described in the Instructions for Authors (https://academic.oup.com/plcell) is: Robert J. Schmitz (schmitz@uga.edu).
